# Evaluation of retinal nerve fiber layer thickness in migraine

**Published:** 2013

**Authors:** Rana Sorkhabi, Somaiyeh Mostafaei, Mohammadhosein Ahoor, Mahnaz Talebi

**Affiliations:** 1 Associate Professor, Department of Ophthalmology, Nikookari Eye Hospital, Tabriz University of Medical Sciences, Tabriz, Iran; 2 Resident, Department of Neurology, Imam Reza Hospital, Tabriz University of Medical Sciences, Tabriz, Iran; 3 Assistant Professor, Department of Ophthalmology, Nikookari Eye Hospital, Tabriz University of Medical Sciences, Tabriz, Iran; 4 Associate Professor, Department of Neurology, Neuroscience Research Center, Tabriz University of Medical Sciences, Tabriz, Iran

**Keywords:** Migraine, Retinal Nerve Fiber Layer Thickness, Optical Coherence Tomography

## Abstract

**Background:**

Headache is one of the most disturbing symptoms with common neurological signs. Variations in optic nerve perfusion quality or retinal microcirculation may end up in ganglion cell damage in patients with migraine.

**Methods:**

Sixty patients diagnosed with migraine and thirty normal individuals were evaluated in groups including migraine with aura, migraine without aura and controls. Retinal nerve fiber layer (RNFL) thickness was measured using stratus optical coherence tomography (OCT™) and then was compared in case and control groups.

**Results:**

RNFL thickness was only significantly thinner in nasal quadrant in migraineurs compared to the control group. Other parameters showed no difference between the two groups and besides there was no statistically difference between the two migraine subgroups.

**Conclusion:**

Given the significant difference in nasal quadrant RNFL thickness between the migraineurs and normal individuals, we might be able to defend the retinal blood flow decrease theory in migraine; however, multicentre studies with larger samples seem mandatory.

## Introduction

Migraine is one of the most common debilitating diseases in the world.^1^ Migraine is divided into two major groups: migraine with aura or classic migraine and migraine without aura or common migraine. Two subgroups are the same in the headache criteria, and the major difference between them is attacks of reversible focal neurologic symptoms in the classic type.^1^ The pathophysiology of migraine has yet to be known. Focal decrease in the regional cerebral blood flow reported in migraine attacks particularly in migraine with aura which starts in posterior circulation.^1^ Decreased blood flow and vasospasm is often confined to one hemisphere.^2^ In rare cases, hypoperfusion starts from other parts of brain or even retina.^3, 4^ Retinal infarctions caused by retinal artery occlusions have been reported in migraine patients.^3, 5^ In addition, cerebral vasospasm is transient; yet recurrent migraine attacks could lead to structural abnormalities. Whether similar changes take place in retina in migraine with or without aura is a subject of uncertainty and few studies have addressed the issue. Alterations in quality of perfusion in optic nerve head microcirculation or even retina may lead to ganglion cell death in migraine patients.^6^

Retinal nerve fiber layer (RNFL) thickness measurements can be used as an index to assess ganglion cell and retinal nerve fiber damages. With the introduction of optic coherence tomography (OCT), the RNFL thickness measurements with 8-10 micrometers scale sensitivity have been possible.^7^ OCT is a reliable and reproducible technique in RNFL thickness measurement. It is a noninvasive, noncontact imaging technique similar to ultrasound imaging except that it uses infra-red wavelengths for 820 nanometers.^8^

This study aimed to quantitatively evaluate and compare RFNL thickness in the prepapillary and macular areas, which was characterized with average thickness, in patients diagnosed with migraine (with/without aura) and control subjects.

## Materials and Methods

This was a prospective, comparative and descriptive study which was done on a group of patients with migraine (study group) and a group of age-matched normal subjects (control group).

The study adhered to the tenets of Declaration of Helsinki and approved by the Ethics Committee of Tabriz University of Medical Sciences. Written informed papers were obtained by all the participants.

The study groups were recruited in a consecutive-if-eligible fashion from the Migraine Clinic of Imam Reza Hospital and controls from the Optometry Services of Nikoukari Eye Hospital in Tabriz, Iran.

According to the results of a pervious study upon RNFL thickness in patients with migraine, with α = 0.05 and power = 90%, the sample size statistically was estimated 25, hence, 60 migraine patients were enrolled in this study accordingly (30 patients in each subgroups), and 30 controls.

All the participants underwent a complete ocular examination including best corrected visual acuity (BCVA) testing with Snellen charts, slit lamp biomicroscopy, dilated fundus examination (DFE) and intraocular pressure (IOP) measurement.

Eyes with retinal and optic disc pathology, glaucoma, dens cataract or corneal opacity which made OCT imaging impossible and eyes previously subjected to intraocular surgery or ocular laser treatment were excluded. Patients with diabetes mellitus, hypertension, cardiovascular and renal disease, history of central nervous system disorders including brain tumors, infarction, encephalitis, epilepsy, Alzheimer's disease, head or eye trauma and any type of headache except for migraine were eliminated from the study.

Participants who met the inclusion criteria were examined by OCT (Stratus OCT-3, Carl Zeiss Meditec Inc., Dublin, CA, USA). All scans were done using an internal fixation target in the OCT device. The fast RNFL scan protocol consisted of three consecutive 360° circular scans with a diameter of 3.4 mm centered on the optic disc.

Parameters including average RNFL thickness in four quadrants were generated automatically in the analysis report. The average and four-quadrant RNFL thickness data (temporal, superior, nasal and inferior) were collected and compared in the both groups.

### Statistical analysis

Analysis was performed using the statistical package for social sciences for windows, (version 16.0 SPSS; Chicago, IL, USA). Data were reported as the mean ± standard deviation (SD). The independent t-test was used to compare the differences between the groups. P-value less than 0.05 statistically considered as a significant level.

## Results

Out of 90 study subjects selected for the study, 60 migraineurs and 30 controls, all met the inclusive/exclusive criteria.

According to the demographic features, there were no statistically significant differences between the migraineurs and control group in sex and age (Table 1).

**Table 1:** Baseline demographic characteristics

Variable	Migraine	Control	P
Number	60	30	-
Age	35 ± 9.2	34.4 ± 9	0.74
Sex	-	-	-
Male	15 (25%)	10 (33.3%)	0.27
Female	45 (75%)	20 (66.7%)	0.27

Out of 60 patients in the migraine group, 30 had migraine with aura and 30 had migraine without aura. The aura was of visual type in all the patients. No significant difference was found between the two migraine subgroups in demographic features and clinical characteristics of headache (Table 2).

**Table 2:** Clinical characteristics of migraine subgroups

Variable	Migraine without aura	Migraine with aura	P
Diagnosis time (year)	7.9 ± 6.4	6.9 ± 5.8	0.510
Frequency of attacks (month)	13.6 ± 10.2	18.6 ± 10.8	0.070
Length of attack (hour)	25.2 ± 31.2	18.5 ± 12.4	0.068
Side of headache	-	-	-
Unilateral	19 (63.3%)	19 (63.3%)	0.610
Bilateral	11 (36.7%)	11 (36.7%)	0.610

RNFL thickness in both migraine and control groups were compared which was significantly lower in nasal quadrant in the eyes of migraine patients (OD: P = 0.01; OS: P = 0.006). There were no statistically differences in the other RNFL thickness parameters between the two groups (Table 3).

**Table 3:** Comparison of retinal nerve fiber layer (RNFL) thickness in migraineurs and control groups

Variable	Case group n = 60	Control group n = 30	P
OD RNFL inferior, µm	126.3 ± 19.3 (40-162)	128.7 ± 14.5 (104-156)	0.540
OD RNFL superior, µm	124.5 ± 18.9 (47-158)	130.2 ± 15 (101-158)	0.150
OD RNFL nasal, µm	77.8 ± 17.3 (53-148)	70.7 ± 8.3 (43-82)	0.010
OD RNFL temporal, µm	70.1 ± 13.6 (42-102)	70.5 ± 11 (54-101)	0.890
OD RNFL average, µm	100.1 ± 11.6 (60-119)	100.5 ± 17.1 (45-133)	0.890
OS RNFL inferior, µm	128.9 ± 17.1 (91-167)	130.1 ± 11.8 (107-155)	0.730
OS RNFL superior, µm	126.7 ± 15.6 (89-162)	127.7 ± 9.6 (106-143)	0.750
OS RNFL nasal, µm	80.6 ± 19.1 (51-134)	71.8 ± 10.5 (59-90)	0.006
OS RNFL temporal, µm	65.6 ± 14.4 (36-116)	68.3 ± 8.9 (54-94)	0.340
OS RNFL average, µm	100.8 ± 9.9 (82-124)	99.5 ± 12.4 (46-117)	0.590

OD RNFL: Retinal nerve fiber layer in right eye; OS RNFL: Retinal nerve fiber layer in left eye.

No statistically significant difference was found between the two migraine subgroups in the all RNFL thickness parameters (Table 4).

**Table 4:** Comparison of retinal nerve fiber layer (RNFL) thickness in migraine subgroups

Variable	Migraine without aura n = 30	Migraine with aura n = 30	P
OD RNFL inferior, µm	124 ± 13 (88-151)	128.5 ± 24.1 (40-162)	0.37
OD RNFL superior, µm	122.7 ± 14.8 (92-146)	126.2 ± 22.3 (47-158)	0.48
OD RNFL nasal, µm	75.2 ± 19.5 (53-148)	80 ± 14.6 (54-111)	0.24
OD RNFL temporal, µm	67.6 ± 13.7 (42-102)	72.6 ± 13.3 (52-102)	0.15
OD RNFL average, µm	98.8 ± 10.6 (76-119)	101.4 ± 12.5 (60-117)	0.37
OS RNFL average, µm	99.5 ± 10 (82-124)	102.1 ± 9.9 (84-120)	0.31
OS RNFL inferior, µm	127.6 ± 17.5 (93-163)	130.2 ± 16.9 (91-167)	0.56
OS RNFL superior, µm	123.6 ± 14 (89-151)	129.8 ± 16.8 (100-162)	0.12
OS RNFL nasal, µm	76.7 ± 19.4 (51-122)	84.4 ± 18.2 (54-134)	0.12
OS RNFL temporal, µm	65.3 ± 15.8 (36-116)	65.9 ± 13 (44-100)	0.88
OS RNFL average, µm	99.5 ± 10 (82-124)	102.1 ± 9.9 (84-120)	0.31

OD RNFL: Retinal nerve fiber layer in right eye; OS RNFL: Retinal nerve fiber layer in left eye.

The correlation between the RNFL thickness and the migraine parameters was compared in patients with migraine with aura and without aura and no significant difference was noted (Table 5).

**Table 5:** Correlation between the retinal nerve fiber layer (RNFL) average thickness and the migraine headache parameters in migraine subgroups

Variable	Headache duration from diagnosis	Headache frequency	Headache severity
RNFL in migraine with aura	P = 0.08	P = 0.13	P = 0.42
RNFL in migraine without aura	P = 0.06	P = 0.68	P = 0.52

## Discussion

One of the proposed mechanisms of migraine and especially migraine with aura is focal brain hypoperfusion in posterior circulation which involves other brain parts and even retina.^6^ RNFL thickness measurements can be used as an index to assess ganglion cell and retinal nerve fiber damages. OCT is a reliable and reproducible technique in the RNFL thickness measurement.^8^ In RNFL topography,^9^ two peridiscal peaks can be seen in the superior and inferior areas. The general method of data illustration begins in temporal quadrants, then goes toward superior, nasal, inferior quadrants and then returns to the temporal; therefore, it is called TSINT Plot.^9^ There are several theories regarding the etiology of optic nerve damage in patients with migraine which include vascular abnormalities such as vasospasm or focal ischemia which both may be either intermittent or chronic.^10^ Supporting the hypothesis of perfusion and vascular retinal variations in migraine, using color Doppler sonography, Kara et al. showed that arterial vessel resistance in central retinal artery (CRA) and posterior ciliary artery (PCA) was higher in patients with migraine in comparison with normal people in periods with no headaches.^11^

The present study used OCT to investigate RNFL thickness in migraine patients and comparing it with normal controls. The results revealed that nasal quadrant RNFL thickness was significantly thinner in migraineurs compared with that of age-matched healthy subjects. Other parameters showed no difference between the two groups.

In study of Martinez et al. in Spain, 70 patients with migraine and 53 normal controls were evaluated by OCT. The mean retinal layer thickness in patients with migraine was in normal range but RNFL thickness in temporal quadrant was significantly reduced in patients with migraine compared to the normal controls.^6^

In the present study, the mean RNFL thickness was significantly reduced in comparison with the normal controls (P = 0.0001). The mean RNFL was 62.6 micrometers in patients with migraine and 70.8 micrometers in the normal cases. However, the thickness difference in superior, inferior and nasal quadrants were not significantly different (P = 0.83, P = 0.15 and P = 0.88, respectively). In the study of Martinez et al., the mean MIDAS (migraine disability assessment score) was also evaluated and was 34.3.^6^. In this study, there was a significant correlation between MIDAS score and mean RNFL thickness (P = 0.0001).

These results differed from the present study in the involved quadrant and also in the relation of headache clinical features with the RNFL thickness which showed no difference in our study. There was no explanation for the discrepancy between the two studies’ results in this study.

Tan et al. in a similar study in Turkey aimed to evaluate the effects of migraine on retina. RNFL thickness in 39 patients with migraine and 25 normal cases was measured using Scanning Laser Polarimetry. They reported there was no statistically significant difference in measurements between both groups.^10^

In this study, the measured RNFL thickness was not significantly different in any of the quadrants in patients with migraine and normal controls. It has been concluded that despite proposed vascular theories, RNFL thickness is not affected in patients diagnosed with migraine.

The difference between this study and ours may be related to the different techniques used for RNFL thickness measurement in the two studies.

Drance et al. in a similar study concluded that retinal vascular changes and RNFL thickness may be affected by normal tension glaucoma in patients with migraine.^12^ In our study, all the cases with any clues of glaucoma were excluded according to the defined criteria. In another study by Gipponi et al. in Italy, RNFL was assessed in patients with migraine. The results showed that RNFL thickness was specially and significantly reduced in superior quadrant in migraineurs (P < 0.05). RNFL was proposed as a simple exam that could be used for evaluation of headache progression over time.^13^ In our study, in contrast to the above study, the RNFL thickness in superior quadrant was not significantly different between the two groups.

## Conclusion

Although the involved quadrants were different between the present study and previous studies, in one point they resemble that migraine can result in focal decrease in cerebral blood flow and especially in retinal circulation.

Considering the significant difference of RNFL thickness in nasal quadrants between patients with migraine and normal cases, there may be a role for retinal infarctions due to occlusion of retinal artery branches in migraine; although larger studies seem mandatory and further evaluation of retinal arteries with angiography is recommended.

Although the significant difference between the clinical features and RNFL thickness was not found in our study, according to Martinez, the proper treatment which reduces migraine attacks frequency and duration may prevent thinning the RNFL thickness, and should be taken into consideration.
